# The Functional Role of Lactoferrin in Intestine Mucosal Immune System and Inflammatory Bowel Disease

**DOI:** 10.3389/fnut.2021.759507

**Published:** 2021-11-25

**Authors:** Ning Liu, Gang Feng, Xiaoying Zhang, Qingjuan Hu, Shiqiang Sun, Jiaqi Sun, Yanan Sun, Ran Wang, Yan Zhang, Pengjie Wang, Yixuan Li

**Affiliations:** ^1^Key Laboratory of Precision Nutrition and Food Quality, Ministry of Education, Department of Nutrition and Health, China Agricultural University, Beijing, China; ^2^Key Laboratory of Functional Dairy, Ministry of Education, Department of Nutrition and Health, China Agricultural University, Beijing, China; ^3^Inner Mongolia Yili Industrial Group, Co., Ltd., Hohhot, China; ^4^Yili Maternal & Infant Nutrition Institute, Beijing, China; ^5^Department of Gastroenterology and Hepatology, University of Groningen and University Medical Center Groningen, Groningen, Netherlands; ^6^Department of Genetics, University of Groningen and University Medical Center Groningen, Groningen, Netherlands; ^7^College of Food Science and Engineering, Gansu Agricultural University, Lanzhou, China

**Keywords:** cytokine, intestinal epithelial cells, immunocytes, lactoferrin, inflammatory bowel disease, intestine mucosal immune system

## Abstract

Inflammatory bowel disease (IBD), encompassing ulcerative colitis (UC) and Crohn's disease (CD), is one of the main types of intestinal inflammatory diseases with intestine mucosal immune disorder. Intestine mucosal immune system plays a remarkable and important role in the etiology and pathogenesis of IBD. Therefore, understanding the intestine mucosal immune mechanism is a key step to develop therapeutic interventions for IBD. Intestine mucosal immune system and IBD are influenced by various factors, such as inflammation, gut permeability, gut microbiota, and nutrients. Among these factors, emerging evidence show that nutrients play a key role in inflammation activation, integrity of intestinal barrier, and immune cell modulation. Lactoferrin (LF), an iron-binding glycoprotein belonging to transferrin family, is a dietary bioactive component abundantly found in mammalian milk. Notably, LF has been reported to perform diverse biological functions including antibacterial activity, anti-inflammatory activity, intestinal barrier protection, and immune cell modulation, and is involved in maintaining intestine mucosal immune homeostasis. The improved understanding of the properties of LF in intestine mucosal immune system and IBD will facilitate its application in nutrition, clinical medicine, and health. Herein, this review outlines the recent advancements on LF as a potential therapeutic intervention for IBD associated with intestine mucosal immune system dysfunction. We hope this review will provide a reference for future studies and lay a theoretical foundation for LF-based therapeutic interventions for IBD by understanding the particular effects of LF on intestine mucosal immune system.

## Introduction

Lactoferrin (LF), an ~80 kDa iron-binding glycoprotein present in most biological fluids (saliva, milk, tears, and mucous secretions), was first identified in 1939, and isolated and purified from human and bovine milk in 1960 (1–4). LF is a safe and reliable natural substance, which is widely used in disease prevention, nutritional supplements, food and drug preservation, and cosmetics. Due to a structure similar to that of serum transferrin (~60%), LF can reversibly bind with ferric (Fe^3+^) ion (5, 6). As an iron transporter, LF protects the nervous system by chelating with iron by reducing oxidative stress and improving iron metabolism (7). Additionally, accumulating evidence demonstrated that LF also possesses antimicrobial, anti-inflammatory, immunomodulatory, anti-carcinogenic, and anti-oxidative activities, thereby highlighting the therapeutic values of this multifunctional protein (8–15).

Bacteriostatic effect of LF is attributed to the binding capacity with free iron, which is an essential element for the growth of bacteria (16). The lack of iron suppresses the growth of *Escherichia coli* (*E. coli*.), an iron-dependent bacteria (17). Conversely, LF may serve as an iron donor to support the growth of some bacteria with lower iron demands, such as *Lactobacillus sp*. or *Bifidobacterium sp*., which is generally considered as a beneficial effect (18, 19). In addition to its antibacterial properties, LF also has both epithelial barrier protection and immunomodulatory properties, which play key roles in the intestine mucosal immune system (20–22). The studies cited above indicate that the physiological functions of LF not only depend on the iron-binding capacity but also on the interaction with molecular and cellular components of both the host and pathogens (23). The intestine mucosal immune system provides a protective barrier against invasion of infectious pathogens and harmful non-self antigens reaching systemic sites within the intestinal tract, and prevents systemic immune responses to commensal bacteria and food antigens (8–10, 13, 14). Inflammatory bowel disease (IBD), mainly divided into ulcerative colitis (UC) and Crohn's disease (CD), is a chronic inflammatory and relapsing disorder of the gastrointestinal tract, in which the interactions among mucosal immune, barrier function, nutrition, and commensal enteric flora are involved (24–28). Accumulating studies report that LF can be considered as a potent anti-inflammatory and immunomodulatory substrate for the prevention and treatment of IBD through regulating intestine mucosal immune response (9, 22, 29, 30). Breakdown of intestinal barrier underpins IBD and other diseases (31). *In vitro* and *in vivo* studies have reported that LF and its derivatives exhibit barrier protection through restoring tight junction (TJ) morphometry, blocking the cleavage of caspase-3, and resuming the drop in transepithelial resistance (TER) in IBD models (30, 32, 33). Furthermore, the LF treatment reduced the secretion and gene expression of tumor necrosis factor alpha (TNF-α), interleukin-8 (IL-8), interleukin-6 (IL-6), and nuclear factor-κB (NF-κB), and signal transducer and activator of transcription 3 (STAT3) signaling pathway activation, both in cultured and Crohn-derived intestinal cells (29, 33–36). It was noted that LF effectively causes dendritic cells (DCs) and macrophages to be tolerogenic phenotype by inhibiting the proliferation of CD4^+^ T cells and enhancing Treg cell differentiation from CD4^+^ T cells in the colon, which is key for tissue homeostasis (37, 38).

Accumulating evidence indicate that LF has been reported to enhance intestinal epithelial cell proliferation, cytokines production, and immune cell functions in counteracting inflammatory processes and maintaining immune homeostasis (9, 22, 29, 30). This review aims to outline the intestine mucosal immune system and the functional role of LF (bovine LF, bLF; human LF, hLF; porcine LF, pLF; LF enzymatic hydrolysate; LF peptide-derivatives) on the intestine mucosal immune system and IBD. We hope this review will lay a theoretical foundation for therapeutic interventions of IBD based on molecular basis and intestine mucosal immune mechanism.

## Intestine Mucosal Immune System

**Figure 1:**
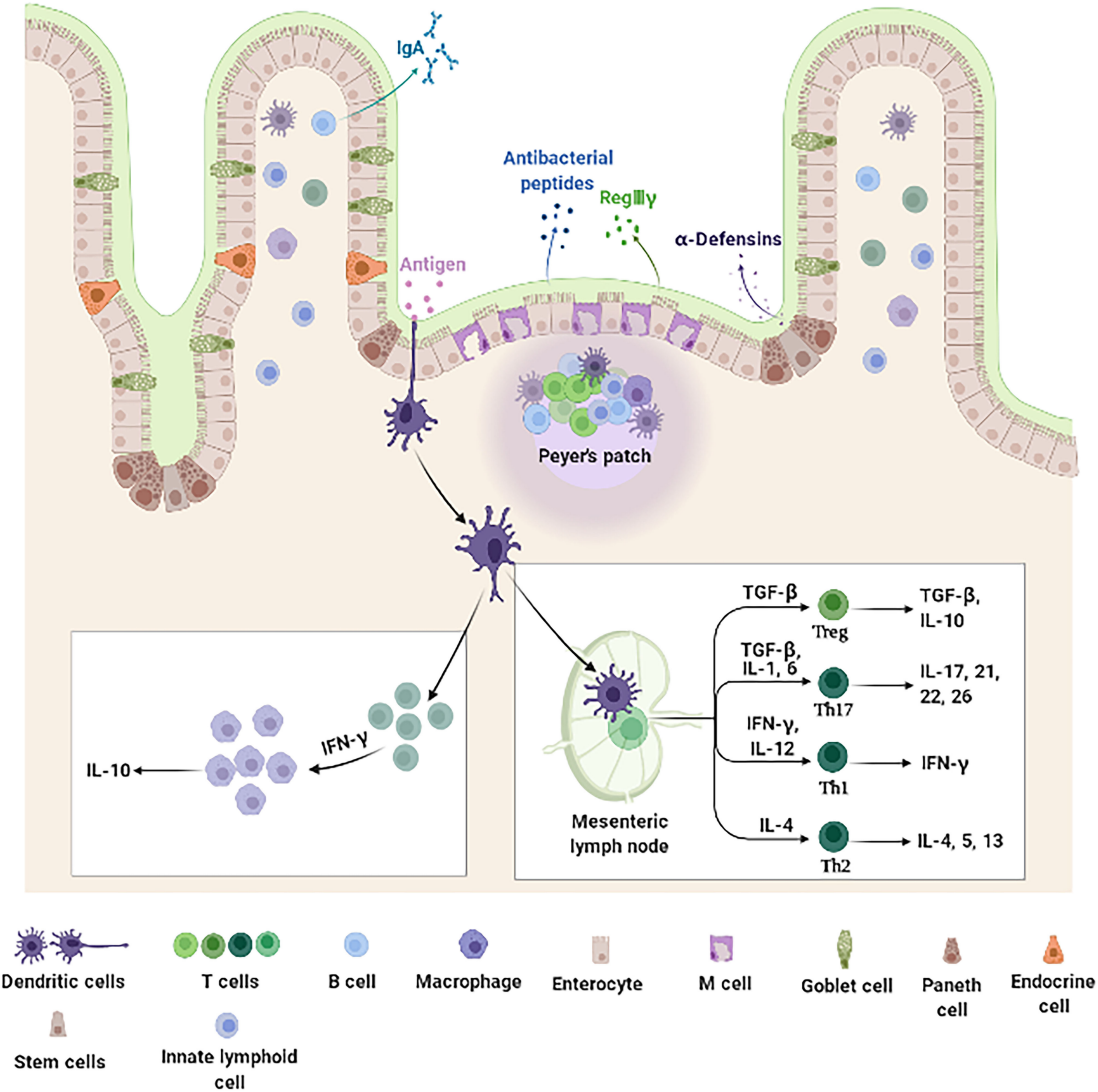
Intestine mucosal immune system landscape. The intestine mucosal immune system is composed of different types of functional cells. Enterocytes, the main functional cells of intestine mucosal immune, are responsible for the absorption of nutrients and water, and also produce antimicrobial peptides (such as RegIIIγ and β-defensin). Paneth cells located at the bottom of the crypt produce amounts of specific antimicrobial peptides (α-defensin). M cells function to sample and transport antigens to immune cells. Goblet cells secrete mucins and promote luminal antigen transfer to DCs. In addition, there are a large number of immune cells distributed in the intestinal epithelium and LP, including DCs, B cells, and T cells. DCs are specialized antigen-presenting cells. After antigens stimulation, they secrete cytokine IFN-γ, which further stimulates monocyte macrophages to secrete IL-10. The DCs that migrate to the MLNs promote the differentiation and maturation of initial T cells. These differentiated and mature T cells can secrete immune factors to participate in intestine mucosal immune system. DCs, dendritic cells; IFN-γ, interferon-γ; IL-10, interleukin-10; LP, lamina propria; M, microfold; MLNs, mesenteric lymph nodes; RegIIIγ, regenerating islet-derived protein IIIγ.

The intestine mucosal immune system, which is mainly composed of intestinal epithelial cells (IECs) and immunocytes (Figure 1), provides a large area for the digestion and absorption of nutrients, serves as a barrier against harmful non-self antigens and infectious pathogens, protects the host against systemic immune responses to commensal bacteria and food antigens, and prevents the trillions of commensal microorganisms living in the gut from reaching systemic sites (39–43). Intestinal epithelial cells not only act as a physical barrier to segregate the intestinal microbiota from the immune cells but also as a coordinator between the intestinal microbiota and immune cells (44). Once the barrier is disrupted, uncontrolled antigens may ingress into the lamina propria (LP) resulting in the release of multiple cytokines, which aggravates the development of inflammation in the intestine (45). Epidemiological observations indeed suggest that patients with IBD have increased intestinal permeability with reduced expression of TJ proteins (46, 47). Additionally, overproduction of proinflammatory cytokines impair the intestinal barrier and induce the accumulation and activation of immune cells, which drive further immune responses and sustain chronic intestinal inflammation in IBD (48, 49). Collectively, IECs and intestinal immunocytes appear of characteristic importance for intestine mucosal immune system and play a key role in pathogenesis of IBD.

### Intestinal Epithelial Cells

The IECs lining the gastrointestinal tract in a single-cell form contain multiple cell types including absorptive columnar epithelial cells, goblet cells, Paneth cells, endocrine cells (ECs), microfold (M) cells, cup cells, and tuft cells, which play important roles in the digestion of food, absorption of nutrients, and protection against microbial infection (48, 50, 51). Additionally, IECs participate in immune activities such as immunoglobulin (Ig)A antibody transportation, antigens uptake, and chemokines and cytokines secretion (44, 52–54). Mounting evidence have demonstrated that IECs produce inflammation and chemokines [such as Interleukin (IL)-18, IL-6, TNF-α, and macrophage chemoattractant protein-1 (MCP-1)] in response to stimulation of intestinal bacteria. Inflammation and chemokines play a vital role in the recruitment, proliferation, activation, and immune response of intestinal immune cells (44, 50, 55). Additionally, IECs directly kill bacteria and regulate the homeostasis of intestinal flora through secreting antibacterial substances, such as defensins, cathelicidins, C-type lectins, ribonucleases (RNases), and S100 proteins (56). Taken together, IECs serve not only as a physical barrier to prevent intestinal bacteria from invading the intestinal mucosa but also as a bridge between innate and adaptive immune systems.

### Intraepithelial Lymphocytes and Lamina Propria Innate Lymphoid Cells

Intestinal innate lymphocytes consist of intestinal intraepithelial lymphocytes (IELs) and LP innate lymphoid cells (ILCs), which are the effector compartments of the intestine mucosal immune system (57). IELs represent one of the largest, non-organized lymphocyte population (58) and constitute one of the most abundant T cell populations of barrier immune cells (59–61). Furthermore, IELs with abundant cytoplasmic granules for cytotoxic activity and expression of effector cytokines [interferon-γ (IFN-γ), IL-2, IL-4, or IL-17] play a crucial role in limiting the dissemination of infectious pathogens and malignant cells and control of infiltration of epithelial surfaces by systemic cells (62, 63). ILCs, identified in the recent years as an important subgroup of natural immune cells, have the dual characteristics of natural immune and acquired immune cells (64). ILCs, which lack T cell receptor (TCR) expression, are innate counterparts of T cells involved in host defense against infection, metabolic homeostasis, tissue repair, and chronic inflammatory diseases by secreting effector cytokines and regulating the functions of other innate and adaptive immune cells (64–66). Under the stimulation of intestinal bacteria, ILCs produce large quantities of cytokines, such as TNF-α, IFN-γ, and IL-17, which in turn stimulate the immune response to eliminate pathogens. However, excessive activation of ILCs in the intestine results in intestinal inflammation and IBD (57, 64). It is noteworthy that the phenotype and function of both IELs and ILCs are disrupted under inflammatory conditions, where they help to exacerbate intestine immune responses (65).

### Dendritic Cells, T Cells, and B Cells

Dendritic cells that reside in the LP of the intestine are the CD103^−^/CX3CR1^+^ subgroup, which patrol among enterocytes and extend dendrites toward the lumen to capture antigens, and then present the antigens to the T cells (67). Additionally, CD103^+^/CX3CR1 subset of DCs can be further divided into two small subgroups, CD11b^+^/CD8α^−^ and CD11b^−^/CD8α^+^, which can migrate into Peyer's lymph nodes and mesenteric lymph nodes (MLNs) (68). DCs continuously migrate through lymphatics to MLNs where they contribute to initial T cell differentiation, maturation, and immune tolerance which is key for intestine mucosal immune system (68).

Intestinal T cells are widely distributed in the Peyer's lymph nodes, MLNs, LP, and intestinal epithelial tissues (69). According to TCR, intestinal T cells are classified into two major subsets, αβT cells and γδT cells, which play a key role in intestinal immune response (70, 71). Furthermore, studies have shown that γδT cells can produce cytokine IFN-γ in response to the stimulation of *E. coli*, followed by IFN-γ stimulating macrophages to release IL-15 which contribute to the accumulation and activation of γδT cells at the site of infection, and anti-infective immunity (72). Additionally, recent evidence have suggested that γδ T cells can secrete IL-17, which recruit neutrophils, macrophages, and natural killer (NK) cells to resist intestinal bacterial pathogens, especially early in infection (73, 74). T cells in the intestinal tract of healthy individuals play a critical role in the process of intestine mucosal immune and intestinal homeostasis (75). However, T cells in IBD patients are excessively active due to intestine mucosal immune dysfunction. Interestingly, reports have found that T cells in the intestine and peripheral blood of patients with CD and UC are significantly elevated, and those T cells in the inflammatory part of the patients show the characteristics of Th17 and Th1 cells, which can secrete IL-17 and IFN-γ inflammation (76). Correspondingly, the treatment of T cells is of considerable importance for the clinical treatment of intestinal inflammatory diseases.

There are a large number of B cells in the intestine; IgA^+^ B cells migrate from Peyer's patches (PPs) to the LP by activation, where they differentiate into IgA-producing plasma cells (77). Under specific immune microenvironment of the intestine, cytokines such as TGF-β and IL-10 are abundant, which promote the differentiation of B cells into secretory IgA plasma cells, and the secreted IgA is transported into the intestinal lumen through IECs to control the invasion of intestinal bacteria by antibody neutralization (78, 79). Therefore, IgA secretory B cells play an extremely important role in the regulation of intestinal flora and the intestinal mucosa defense. Although IgM is the first Ig produced by B cells, the B cells are stimulated by antigens in the germinal center of lymphoid tissues and follicular helper T cells (TFHs), which cause antibody class-switch recombination (CSR) to produce IgG, IgA, and IgE (80).

## Immunomodulatory Role of Lactoferrin in Intestine Mucosal Immune System

**Figure 2:**
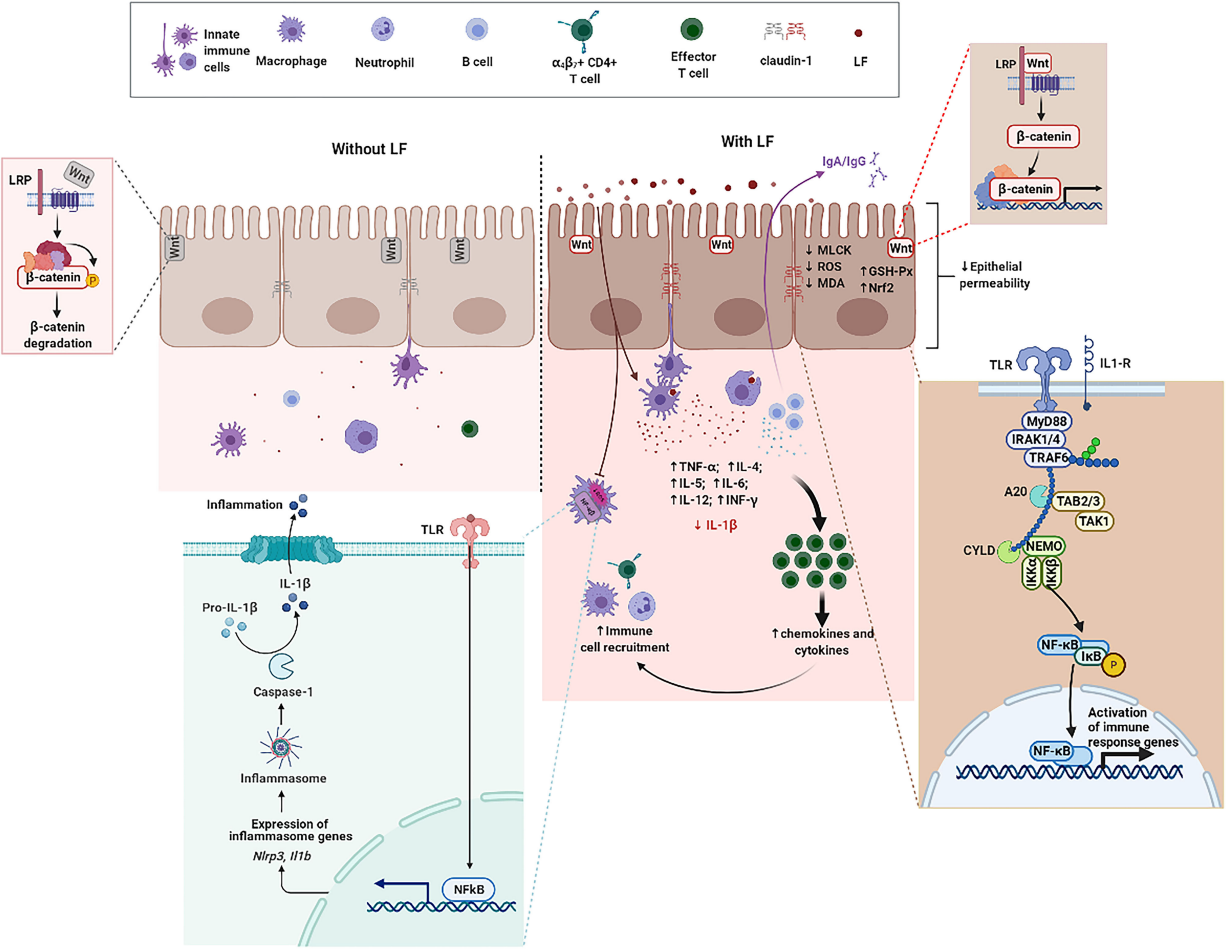
The functional role of LF on intestine mucosal immune system in the context of IBD. Without LF **(Left)**, the intestine mucosal immune system follows the path of autoimmune regulation. The presence of LF **(Right)** promotes TJ between epithelial cells, enhances the expression of β-catenin, and activates the Wnt signaling pathway. LF downregulates the protein abundance of MLCK, reduces the level of ROS and MDA, enhances GSH-Px activity, and upregulates the expression level of Nrf2 in epithelial cells. LF enhances the expression level of TLRs and activates the NF-κB pathway. LF also reduces the secretion and the gene expression of IL-1β, enhances the function of immune cells, promotes the production of cytokines, and promotes the recruitment of immune cells. GSH-Px, glutathione peroxidase; IL-1β, interleukin-1β; LF, lactoferrin; NF-κB, nuclear factor kappa-B; Nrf2, nuclear factor erythroid 2-related factor 2; MDA, malondialdehyde; MLCK, myosin light chain kinase; ROS, reactive oxygen species; TJ, tight junction; TLRs, toll-like receptors.

Lactoferrin, an ~80 kDa single polypeptide chain glycoprotein belonging to transferrin family, is widely present in external secretions (milk, seminal fluid, saliva, tears, and mucous secretions) and in some granules of polymorphonuclear leukocytes (20, 81). The presence of disulfide bonds between cysteine residues in LF partly contribute to the secondary structure comprising 33–34% helices and 17–18% strands (82). The three-dimensional structure of LF consists of two highly homologous lobes, the N- and C-lobes (83). Each lobe further consists of two sub-lobes or domains which have high affinity with single Fe^3+^ (84). Accumulating evidence indicate that LF can regulate the proliferation of IECs, development and maturation of immune cells, and production of cytokines to counteract inflammatory processes and maintain intestine mucosal immune homeostasis in the context of IBD (Figure 2) (85, 86). Antimicrobial activity, modulation of cytokine production, immune cell migration, and the maturation and growth of immune or epithelial cells are partly due to LF interactions with pathogen-associated microbial patterns (PAMPs), glycosaminoglycans, or iron (86, 87). Thus, the functional role and underlying mechanisms of LF on IECs, immune cell response, and cytokine production are overviewed in Table 1 and discussed in the ensuing sections.

**Table 1:** An overview of the properties of lactoferrin in the intestine mucosal immune system.

**Model**	**Source**	**Dose**	**Time**	**Findings**	**References**
*In vitro*, Caco-2 cells/J774A.1 cells	Bovine apo-, native- and holo-LF	5 mg/mL	24 h	Neutralized microbial-derived antigens; Reduced pro-inflammatory effect	(88)
*In vitro*, IPEC-J2 cells	Bovine native-LF	0.1, 0.25, 0.5, 1.0, 1.5, or 3.0 mg/mL	24 h	Alleviated the LPS-induced cellular inflammation; Reduced NF-κB/MAPK pathways; Maintaining cellular barrier integrity and mitigating oxidative stress; Reduced intracellular reactive oxygen species level and malondialdehyde level; Upregulated the glutathione peroxidase activity and the expression of nuclear factor erythroid 2-related factor 2 (Nrf2) protein; Reduced the IL-1β secretion; Downregulated the phosphorylation levels of NF-κB, IκB, p38, and ERK1/2 in LPS-challenged cells	(36)
*In vitro*, Caco-2/TC7 cells	Bovine LF	0.5, 1, 2, 5 or 10 mg/mL	24 h	Altered the expression of TLR2, TLR4, and TLR9 receptors; Reduced expression levels of TLR4; Maintaining redox homeostasis	(89)
*In vivo*, zebrafish	Bovine LF	0, 0.5, 1, or 1.5 g/kg	3 d	Enhanced the neutrophil migration and intestinal mucosal barrier functions related genes expression; Improved performance against bacterial infection	(90)
*In vivo*, mice	Bovine holo-LF	0, 50, 500, or 5000 μg/day	7 d	Enhanced level of total and specific IgA, protein expression of a-chain and pIgR, mRNA transcripts of a-chain, IL-2 and IL-5, and level of plasma corticosterone	(91)
*In vivo*, mice	Bovine LF	2.0% bLF in water or diet	84 d	Improved fecal score, lesions in the colon, and body weight loss	(92)
*In vivo*, rats	Bovine LF	0.5 g/kg/d	18 d	Enhanced small-bowel sIgA concentrations and tight junction proteins expression; Reduced intestinal permeability; Supported intestinal barrier integrity; Protected against bacterial infections	(93)
*In vivo*, neonatal piglet	Bovine LF	130, 367 or 1300 mg/kg BW/d	14 d	Altered the capacity of MLNs and spleen immune cells; Initiated immune responses in immunologically challenged neonates	(94)
*In vivo*, piglet	Bovine LF	0.4, 1.0, or 3.6 g/L	14 d	Enhanced intestinal crypt proliferation and crypt depth; Enhanced β-catenin mRNA expression	(95)
*In vivo*, piglet	Recombinant human LF	2, 11, or 20 mg/g	30 d	Reduced diarrhea; Boosted humoral immunity, Th1, and Th2 cell response; Improved intestinal morphology; Activated the immune-related genes expression	(96)
*In vivo*, human	Recombinant human LF	1500 mg/d	90 d	Did not reduce inflammation and immune activation	(97)
*In vitro*, bacteria	Peptide-derived from Bovine LF	0.3-150 mg/mL	16 to 20 h	Attenuated the LPS induced immune disorders; Sustained the balance of CD3^+^/CD8^+^ T cells, B cells and NK cells; Activated cellular defense and stimulated B cells to secrete certain IgG	(98)
*In vivo*, mice	Peptide-derived from Porcine LF	0, 2.5, 5, or 10 mg/kg	7 d	Balanced Th1 and Th2 response; Triggered cellular defense mechanisms and induced B cells to produce antibodies to defend against LPS stimulation	(99)

### Lactoferrin Performs Protection of Intestinal Epithelial Cells

Many studies have suggested that LF has anti-inflammatory effects, but the protective effect on small IECs is still poorly understood. Hu et al. took the intestinal porcine epithelial cell line-J2 (IPEC-J2) as the research model to investigate the protective effects and underlying mechanisms of bLF on lipopolysaccharides (LPS)-challenged IPEC-J2 cells *in vitro*. Treatment with bLF resulted in reduced cell permeability, enhanced Claudin-1 protein abundance, and inhibition of myosin light chain kinase (MLCK) protein abundance in LPS-challenged cells (36). Mounting evidence demonstrated that sIgA and the polymeric immunoglobulin receptor (pIgR) play a pivotal role in immune homeostasis by limiting the access of microbial and environmental antigens into the body, maintaining the integrity of the epithelial barrier, and shaping the composition of the commensal microbiota (100–102). bLF supplementation enhances the production of sIgA in small-bowel, supports intestinal barrier integrity by upregulating TJ protein express, and protects intestine from bacterial infections (93). Additionally, previous study reported that formula supplemented with bLF enhanced intestinal crypt proliferation and crypt depth. Furthermore, jejunal crypt cells isolated by using laser capture microdissection (LCM) had enhanced β-catenin mRNA expression, which suggests that the Wnt signaling may partly be involved in cell proliferation induced by bLF (95). Tanaka et al. found that oral administration of bLF protected the mucus barrier overlying the intestinal epithelium against dextran sodium sulfate (DSS)-mediated damage. Notably, bLF supplementation led to the inhibition of cell division in intestinal crypts, which in turn affected carcinogenesis in the colon of LPS-challenged mice (92). Despite mounting basic researches on LF, which is abundant in mammalian colostrum and milk, very little is known about the effects of metal saturation (iron-depleted, iron-saturated, and manganese-saturated forms) of LF on intestinal barrier function. For this goal, researchers used Caco-2, a human intestinal epithelial cell line, to investigate the effects of bLF with iron and manganese saturation on the health of the host. Results indicated that no changes of TJ proteins were observed in response to bLF metal saturation status. Notably, different bLF forms markedly suppressed the pro-inflammatory response in macrophage through binding and neutralizing LPS (88). Additionally, LF was also able to neutralize microbial-derived antigens, thereby potentially reducing their pro-inflammatory effect (103). The effect of bLF as a regulator of intestinal innate immunity and oxidative stress on IECs was investigated in a previous study. Innate immune Toll-like receptors (TLRs) mRNA expression, lipid peroxidation, and protein carbonyl levels were determined in enterocyte-like Caco-2/TC7 cells incubated with bLF for 24 h. Results demonstrated that bLF seemed to maintain redox homeostasis and modulate inflammatory response *via* activation of TLRs when exposed to LPS (89). Additionally, LF reduced intracellular ROS level and malondialdehyde (MDA) level as well as upregulated glutathione peroxidase (GSH-Px) activity and the expression of nuclear factor erythroid 2-related factor 2 (Nrf2) protein (36).

### Lactoferrin Modulates Immune Cell Function and Cytokine Production

Dietary bLF alters the capacity of MLNs and spleen immune cells in response to stimulation, providing a protective role for LF in the initiation of immune responses in these immunologically challenged neonates (94). It is noted that recombinant human LF (rhLF) secreted by transgenic cattle was used to investigate the immunomodulatory effects of rhLF on the systemic and intestinal immune system in piglets, which are good models widely used in infant nutrition study. Results showed that rhLF milk significantly reduced diarrhea, boosted humoral immunity, Th1 and Th2 cell response, improved intestinal morphology, and activated the transcription of important immune-related genes expression (96). Study on incorporation bLF into soybean meal-based diet demonstrated that 1.5 g/kg bLF supplemented to soybean meal reduced the neutrophils in the intestine when compared with control. Likewise, bLF supplementation enhanced the neutrophil migration and intestinal mucosal barrier functions related to genes expression. These findings suggested that bLF acts as an intestinal anti-inflammatory agent and improves performance against bacterial infection (90). These results indicate a potential role of LF in intestine mucosal immune. Ingestion of soybean meal resulted in intestinal inflammation which is a harmful condition in fish. Interestingly, peptide derived from bLF is also capable of conserving the biological activity (98). LFP-20, a twenty-amino acid antimicrobial peptide in the N terminus of pLF, has been reported to modulate inflammatory response in colitis (99). Pre-treatment with LFP-20 attenuated the LPS-induced immune disorders in ileum and sustained the balance of CD3^+^/CD8^+^ T cells, B cells, and NK cells. Furthermore, LFP-20 facilitated a balanced Th1 and Th2 response. Of note, LFP-20 activated the cellular defense and stimulated the B cells to secrete certain IgG (99). Although mounting researches have focused on exogenous LF, there is little information available regarding the expression of endogenous LF in response to bacterial infection. Previous study indicated that distribution of LF in mice intestine during *E. coli* K88 infection was upregulated in duodenum, ileum, and colon, but reduced in jejunum, by using PCR and immunohistology staining. These data pave the way for a better understanding of the key role of LF in intestine mucosal immune (104). A large number of studies have reported that LF regulates mucosal immune and targets the mechanism that induce inflammation (105). A clinical trial on rhLF conducted with 54 human immunodeficiency virus-infected participants with viral suppression demonstrated that no differences were observed in IL-6, D-dimer levels, monocyte/T-cell activation, mucosal integrity, or intestinal microbiota diversity when compared with controls (97).

Under physiological conditions, bLF supplementation led to the upregulation of sIgA, the protein expression of α-chain and pIgR, and the mRNA expression of α-chain, IL-2, and IL-5 (91). The result suggested that bLF contributed to maintain intestinal homeostasis through an interleukins profile that favored the IgA antibody response (91). Recently, studies indicated that LF was essential for the development of the early stages of B cells in mice by regulating the microenvironment of bone marrow stroma through C-X-C motif chemokine ligand 12 (CXCL12) release (106). Correspondingly, a previous study found that bLF treatment reduced the IL-1β secretion and mRNA expression, and downregulated the phosphorylation levels of NF-κB, IκB, p38, and ERK1/2 in LPS-challenged cells (36). Interestingly, peptide derived from bLF is also capable of conserving the biological activity (98). LFP-20, a twenty-amino acid antimicrobial peptide derived from pLF, has been reported to modulate inflammatory response in colitis (99). Pre-treatment with LFP-20 facilitated a balanced Th1 and Th2 response, which is consistent with the modulation of Th1 cytokines (IL-12p70, IFN-γ, and TNF-α) and Th2 cytokines (IL-4, IL-5, and IL-6) (99).

## Functional Role of Lactoferrin in Inflammatory Bowel Disease

IBD, mainly divided into UC and CD, is a chronic inflammatory and relapsing disorder of the gastrointestinal tract in which the interactions among mucosal immune, barrier function, nutrition, and commensal enteric flora are involved (24–28, 107). IBD has become a global disease with rapidly increasing incidence and prevalence, and been diagnosed in developed and developing countries in both men and women (108–111). LF, a multifaceted milk protein, is considered as a potent anti-inflammatory and immunomodulating substrate for protecting mucosa against infections and inflammation (29). Accumulating studies report that LF can be considered as a potential therapy for the prevention and treatment of IBD based on the beneficial effects of LF in inhibiting invasion or adhesion of bacteria or modulating/boosting mucosal immune system (Figure 2) (9, 22, 30).

Oral administration offers the most convenient way for supplementing LF, which is considered as a new clinical nutrition strategy for the treatment of IBD (112, 113). As expected, LF ingested through diet, water, or perorally is degraded rapidly by enzymatic hydrolysis in the gastrointestinal tract, which causes undesirable loss of its functional properties (20). Therefore, high amounts, frequent dosing, or an appropriate delivery system may improve its bioavailability (112). Nevertheless, previous study showed that much more undigested LF enters the intestine when it is administered by gavage (20). Two studies that administered LF by gavage to DSS-treated mice found significantly less damage in the colon of the LF groups (34, 114). As matter of fact, antimicrobial peptides such as lactoferricin and lactoferrampin are generated during gastric and intestinal digestion stages (115–119). LF or its derived fragments in high amounts binding to LF receptors in intestinal mucosa and gut-associated lymphatic tissue-related cells would modulate cytokine/chemokine production and immune cells function. Additionally, LF administered to DSS-treated mice *via* intracolonic injection during the DSS treatment period markedly reduced damage in the colon than the controls (120).

### Bacteriostatic Properties of Lactoferrin in Inflammatory Bowel Disease

Intestinal microbiota plays a key role in the development and maintenance of IBD (121). Therefore, manipulation of the gut microbiota may represent a target for IBD therapy (122). LF has broad spectrum antibacterial properties against a wide range of pathogenic bacteria including gram-positive bacteria and gram-negative bacteria (116, 123). A previous study was conducted to investigate the ability of bLF to modulate the interactions between the adherent-invasive *E. coli* strain LF82 (ileal Crohn's strain) and Caco-2 cells. Scanning electron microscopy, transmission electron microscopy, and light microscopy revealed that bLF prevents invasion of *E. coli* strain LF82 by binding with the bacteria type 1 pili (29). Recent study reported that bLF-treated DSS-challenged mice turn the *Muribaculaceae*/*Lachnospiraceae* intestinal type into *Akkermansiaceae*/*Bacteroidaceae* intestinal type in colitis mice. This result indicates a direction toward treating colitis by changing the structure and composition of intestinal microbiota. Additionally, metabolomics results demonstrated that bLF changed purine metabolism when compared with DSS-challenged mice (30). However, the underlying mechanisms responsible for the antibacterial properties of LF have not been completely elucidated (124). Accumulating evidence demonstrated that antibacterial activity of LF not only depends on the iron binding capacity but also on serine protease and the permeability of the bacterial cell membrane destruction (17–19, 125–128).

### Epithelial Barrier Protection of Lactoferrin in Inflammatory Bowel Disease

The intestinal epithelial barrier integrity is vital to protect the intestinal cells from microbes, and intestinal barrier dysfunction underpins IBD and other diseases (31, 129–131). *In vitro* studies reported that bLF exerts a protective function toward intestinal barrier disorder (32, 33). Hu et al. took TNF-α-challenged HT-29/B6 cells to elucidate the protective properties of bLF on intestinal epithelial barrier and found that bLF restored TJ morphometry and almost completely blocked the cleavage of caspase-3 induced by TNF-α. Additionally, the results of this study demonstrated that bLF treatment resumed the drop in TER and Claudin-8 down-regulation when HT-29/B6 or T84 cells were challenged with *Yersinia enterocolitica* infection (32). In another study conducted by Zhao et al. it was found that bLF significantly enhanced the expression of Claudin-1, Occludin, and ZO-1 at both the mRNA and protein levels (33). Additionally, *in vivo* studies demonstrated that bLF administration ameliorated the severity of DSS or azoxymethane (AOM)-induced colitis as reflected by reduced body weight loss, decreased colon shortening, and reduced myeloperoxidase (MPO) activity (30, 92). Moreover, protein abundance of Claudin-1, Occludin, ZO-1, and regenerating islet-derived protein IIIγ (RegIIIγ) in the colon were enhanced by bLF treatment when compared with the DSS group (30). It was also noted that oral administration of a bovine lactoferricin–lactoferrampin (LFCA)-encoding *Lactococcus lactis* strain prevented DSS-induced colitis through enhancing the protein abundance of ZO-1, E-cadherin, and Claudin-2 (132).

### Anti-inflammatory Properties of Lactoferrin in Inflammatory Bowel Disease

In addition to its epithelial barrier protection properties, LF also has anti-inflammatory properties (29, 32, 90, 92, 133). LF affects type 1 interferon expression or immune cell function (13, 134). Growing evidence reported that LF inhibits TNF-α, IL-8, IL-6, and NF-κB signaling pathway activation both in cultured and Crohn-derived intestinal cells (29, 33, 34). In experimental colitis, LF administration leads to a significant reduction in TNF-α, IL-1β, and IL-6, and an increase of IL-4 and IL-10 (35). Furthermore, LF administration ameliorates DSS-induced intestinal inflammation in mice by suppressing NF-κB signaling pathway activation (135). In particular, results from previous study showed that apo-bLF was more efficient than the holo form in decreasing MPO, IL-1β, and TNF-α synthesis in trinitrobenzenesulfonic acid (TNBS)-induced colitis in rats and dextran sulfate (DSS)-induced colitis in mice (34, 38, 136). Moreover, bLF has been considered as a negative regulator for IL-6 production in *in vitro* and *in vivo* studies as well as in clinical trials (133, 137–142). Interestingly, bLF was also found to interfere with STAT3 activation pathways both in IL-6-dependent and IL-6-independent modes (138, 143–145).

### Immune Cell Modulation of Lactoferrin in Inflammatory Bowel Disease

Studies have revealed that uncontrolled activation of intestine immune cells and pathogenic immune cells circuits contribute to the onset and development of IBD (48, 146). LF supplementation enhances the expression of CD80, CD83, and CD86, and the production of proinflammatory cytokines of monocyte-derived dendritic cells, which indicate this type of cell maturation (124). Furthermore, LF effectively causes DCs to be tolerogenic by suppressing CD4^+^ T cells proliferation and enhancing Treg cell differentiation from CD4^+^ T cells in the colon, when compared with the DSS group (37, 38). Recently, LF has been reported to promote the macrophage shift from inflammatory to tolerogenic phenotype, which is key for tissue homeostasis (133). Consistently, VEN-120, a recombinant human LF, reverses severe inflammation in both the DSS-induced colitis model and the TNFΔARE/+ model of ileitis by increasing Treg cells in LP. *In vitro* study confirmed that CD4^+^ T cells treated with LF upregulates Treg genes and Treg populations (114). Overall, the studies cited above indicate that LF and LF-derived peptide fraction can be considered as an effective clinical nutrition strategy for the treatment or prevention of IBD.

## Conclusions

The intestine mucosal immune system is a complex network composed of lymph nodes, LP, and epithelial cells, which provides a barrier to separate the intestinal luminal contents from the internal environment, and plays an essential role in a perfect immune response mechanism and a strict immune regulation mechanism. LF, an iron-binding protein expressed in most biological fluids, has been considered as a potent antimicrobial, anti-inflammatory, and immunomodulatory substrate for modulating/boosting intestine mucosal immune system and protecting the intestine against IBD and other diseases. Owing to the various microbial and host targets of LF, understanding the mechanisms of action of LF in the intestine mucosal immune and IBD is a challenge. Therefore, the underlying mechanisms are still under investigation and further studies are needed. Currently, LF interaction with PAMPs, glycosaminoglycans, or iron as well as nucleus seem to be the most reasonable mechanisms contributing to change in the structure and composition of the intestinal microbiota, maintenance of intestinal epithelial barrier integrity, balance between proinflammatory and anti-inflammatory cytokines production, and immune cell function modulation, which are critical for intestine mucosal immune system and IBD. Therefore, understanding the molecular basis and intestine mucosal immune mechanism is a key step to develop therapeutic interventions, and provides a new target for the treatment of IBD associated with intestine mucosal immune system dysfunction.

## Author Contributions

Writing—review and editing were carried out by NL, GF, XZ, QH, SS, JS, YS, RW, YZ, and PW. Supervision was done by YL. All authors have read and agreed to the published version of the manuscript.

## Funding

This work was supported by the National Natural Science Foundation of China (Nos. 31901625, 32130081, 32000082, and 31625025), State Key Laboratory of Animal Nutrition (2004DA125184F1909), Huhhot Science & Technology Plan (No. 2020-Ke Ji Xing Meng-National Innovation Center-3), and the 111 Project (B18053).

## Conflict of Interest

GF, XZ, and JS were employed by Inner Mongolia Yili Industrial Group, Co., Ltd. The remaining authors declare that the research was conducted in the absence of any commercial or financial relationships that could be construed as a potential conflict of interest.

## Publisher's Note

All claims expressed in this article are solely those of the authors and do not necessarily represent those of their affiliated organizations, or those of the publisher, the editors and the reviewers. Any product that may be evaluated in this article, or claim that may be made by its manufacturer, is not guaranteed or endorsed by the publisher.
